# The effects of steamed ginger ethanolic extract on weight and body fat loss: a randomized, double-blind, placebo-controlled clinical trial

**DOI:** 10.1007/s10068-019-00649-x

**Published:** 2019-10-11

**Authors:** Soo-Hyun Park, Su-Jin Jung, Eun-Kyung Choi, Ki-Chan Ha, Hyang-Im Baek, Yu-Kyung Park, Kap-Hoon Han, Soon-Yeon Jeong, Jung-Hee Oh, Youn-Soo Cha, Byung-Hyun Park, Soo-Wan Chae

**Affiliations:** 1 Clinical Trial Center for Functional Foods, Chonbuk National University Hospital, Jeonju-si, Republic of Korea; 2 Department of Food Science and Human Nutrition, Chonbuk National University, Jeonju-si, Republic of Korea; 3 Healthcare Claims and Management Incorporated, Jeonju-si, Republic of Korea; 4 Department of Pharmaceutical Engineering, Woosuk University, Wanju-Gun, Republic of Korea; 5 Healthy Local Food Branding Agency, Wanju-Gun, Republic of Korea; 6 Department of Biochemistry, Chonbuk National University Medical School, Jeonju-si, Republic of Korea; 7 Department of Pharmacology, Chonbuk National University Medical School, Jeonju-si, Republic of Korea

**Keywords:** Steamed ginger ethanolic extract, 6-Shogaol, Body fat, Weight, Clinical trial

## Abstract

Steamed ginger ethanolic extract (SGE) is a product with a high 6-shogaol contents and is thought to be more potent than other ginger products. We conducted a 12-week, randomized, double-blind, placebo-controlled clinical trial to determine the effects of SGE on weight and body fat loss. Eighty healthy obese participants were recruited and randomly divided into the SGE and placebo groups. The outcome measures comprised indicators of efficacy (body weight, body mass index, body composition, and blood markers) and safety. Following the supplementation period, mean body weight, body mass index, and body fat level were significantly lower in the SGE group than in the placebo group. No clinically significant changes were observed for any safety parameter. These results suggest that SGE is a potent anti-obesity agent that does not cause significant side effects. Therefore, SGE supplementation combined with lifestyle modification could be effective in the management of body weight and fat mass.

**Electronic supplementary material:**

The online version of this article (10.1007/s10068-019-00649-x) contains supplementary material, which is available to authorized users.

## Introduction

Obesity is caused by energy input exceeding energy output over a prolonged period. Obesity leads to many health problems, including hypertension (Jayedi et al., 2018), hyperlipidemia (Klop et al., 2013), diabetes mellitus (Caspard et al., 2018), cardiovascular disease (Kotsis et al., 2018), arthritis (Van Raemdonck et al., 2018), atherosclerosis (Lovren et al., 2015), and cancer (Ottaiano et al., 2018).

Diet control and physical activity are the most common methods of preventing obesity. Today, there is a growing interest in reducing body weight through the use of drugs or supplements in combination with diet control and exercise. However, the dietary supplements known to reduce body weight may have side effects, as has already been reported for anti-obesity drugs such as appetite suppressants (Krentz et al., 2016). Therefore, it is necessary to develop healthy functional foods that have anti-obesity effects with no side effects.

Ginger (*Zingiber officinale*) is a perennial herbaceous plant widely distributed in India, East Asia, and Southeast Asia and is used as a pungent flavoring spice. Ginger has been shown to have antioxidant (Danwilai et al., 2017), anti-inflammatory (Zehsaz et al., 2014), anti-cancer (Pashaei-Asl et al., 2017), neuroprotective (Saenghong et al., 2012), anti-emetic (Saberi et al., 2014), and anti-hyperglycemic effects (Maharlouei et al., 2018). In addition, anti-obesity effects of ginger have been reported in many preclinical and clinical studies (Maharlouei et al., 2018; Misawa et al., 2015; Nammi et al., 2009; Wang et al., 2017).

The nutraceutical and pharmaceutical functions of ginger result primarily from its bioactive phytochemicals, especially the pungent principle gingerols and their dehydrated products, shogaols (Semwal et al., 2015). Gingerols are structurally unstable because they contain a β-hydroxyl group. They are converted to shogaols by dehydration of the β-hydroxyl ketone skeleton through drying, blanching, pasteurization, and extraction, and the most common dehydration product is 6-shogaol (Bhattarai et al., 2001; 2007; Jung et al., 2018). According to prior studies, 6-gingerol and 6-shogaol are major bioactive compounds, and some studies have demonstrated that 6-shogaol is superior to 6-gingerol in terms of its antioxidant (Dugasani et al., 2010), anti-inflammatory (Dugasani et al., 2010; Ho and Chang, 2018), anti-emetic (Jin et al., 2014), and anti-cancer activities (Wang et al., 2015).

Steamed ginger ethanolic extract (SGE) is a ginger product in which the 6-shogaol content has been increased through steaming and ethanol extraction. SGE is expected to be more potent than other ginger products due to the α,β-unsaturated ketone structure of its side-chain (Kou et al., 2018). In a previous study, SGE was reported to inhibit hepatic lipid accumulation by suppressing the expression of SREBP1c (*Srebpf1*), a lipogenic transcription factor. SGE treatment also significantly increased markers of body fat reduction, including weight loss, in an animal model fed a high-fat diet (Kim et al., 2018).

Although the anti-obesity effects of ginger have been well documented in animal and human studies, the effects of 6-shogaol-enriched ginger extract have only been investigated in preclinical and clinical studies. Therefore, in this study, we investigated whether SGE could be effective for weight and body fat loss in participants with obesity.

## Materials and methods

### Study design

This study was a 12-week, randomized, double-blind, placebo-controlled clinical trial with a parallel-group design that investigated the effects of SGE supplementation on weight and body fat loss. The randomization scheme was generated by a computerized procedure. The randomization sequence and allocation were concealed from all participants, investigators, and research staff until completion of the study. The study was conducted according to the Helsinki Declaration and the Guideline for Good Clinical Practice by the International Conference on Harmonization (ICH GCP). The study protocol and informed consent form were reviewed and approved by the Institutional Review Board (IRB) of Chonbuk National University Hospital. Each participant provided written informed consent before the study began. The study was conducted from 2017 to 2018 at the Clinical Trial Center for Functional Foods at Chonbuk National University Hospital. During the 12-week intervention period, participants were asked to continue their usual diets and not to consume any other functional foods or dietary supplements. Participants were asked to report their capsule compliance, along with any adverse events or changes in lifestyle or eating patterns. Efficacy and safety were evaluated before and after the intervention period.

### Test supplement

The SGE supplement was prepared by the Healthy Local Food Branding Agency (Wanju, Korea). Steamed and dried ginger was subjected to ethanol extraction. For standardization, the sample was extracted with methanol and analyzed by high-performance liquid chromatography (HPLC) at a detection wavelength of 230 nm, with 6-shogaol as the marker compound (Fig. S1). The 6-shogaol concentration of the SGE was standardized to 5.89–8.83 mg/g. The supplement was administered in capsules containing 100 mg SGE and a diluting agent. The placebo capsules were composed primarily of microcrystalline cellulose, caramel color, and ginger flavor and were matched for energy content, flavor, appearance, and dosage (Table 1). All participants were instructed to take two capsules per day. The SGE and placebo capsules were packaged in an indistinguishable manner and were labeled with the study participant’s number.

**Table 1:** Compositions of investigational products

Component	Content (%)	
	Placebo supplement	SGE supplement
SGE	–	25
Microcrystalline cellulose	98	73.6
Caramel color	1.5	1.1
Ginger flavor	0.5	0.3
Total	100	100

### Participants

Females from Korea who were obese (25 ≤ body mass index (BMI) < 30 kg/m^2^) according to the Asia–Pacific guidelines and had not been diagnosed with any other diseases were included in this study (Consultation, 2004). Eighty participants met the study criteria and were randomly divided into two groups (n = 40 each), to be given either SGE (200 mg) or placebo.

The exclusion criteria for the study were as follows: (1) significant variation in body weight (greater than 10%) in the previous three months; (2) treatment with anti-obesity drugs or hypolipidemic drugs within the previous four weeks; (3) history of cardiovascular disease, including arrhythmia, heart failure, myocardial infarction, or use of a pacemaker; (4) history of conditions that could interfere with the test products or impede their absorption, such as gastrointestinal disease or prior surgery; (5) participation in another clinical trial within the previous 2 months; (6) abnormal hepatic function; (7) history of renal disease; (8) anti-psychotic drug therapy within the previous 2 months; (9) laboratory test results and medical or psychological conditions that could interfere with successful participation in the study, as judged by the investigators; (10) pregnancy or breastfeeding; (11) history of alcohol or substance abuse; and (12) allergy or hypersensitivity to any of the ingredients in the test supplements.

### Efficacy outcome measures

The efficacy assessment was based on anthropometric parameters (body weight, BMI, waist circumference, and hip circumference), body composition (body fat mass, percent body fat, and fat-free mass), and blood markers [total cholesterol, triglyceride, high density lipoprotein (HDL)-cholesterol, low density lipoprotein (LDL)-cholesterol, adiponectin, and leptin levels]. Body composition was measured by dual-energy X-ray absorptiometry (DEXA) (Discovery W, Hologic, Marlborough, MA, USA). Total cholesterol, triglycerides, and HDL-cholesterol levels were measured on a Hitachi 7600-110 analyzer (Tokyo, Japan). The LDL-cholesterol level was calculated by the Friedewald formula (Friedewald et al., 1972). Adiponectin was measured with an ELISA kit (Biovendor, Brno, Czech Republic), and leptin was measured with an RIA kit (LINCO Research, St. Charles, MO, USA).

### Safety outcome measures

The safety assessments comprised electrocardiography, vital signs measurements (blood pressure and pulse rate), and laboratory tests (WBC, RBC, Hb, Hct, platelet count, ALP, gamma-GT, AST, ALT, total bilirubin, total protein, albumin, BUN, creatinine, glucose, creatine kinase, and LDH). The WBC, RBC, Hb, Hct, and platelet counts were measured on a System XE-5000TM (Sysmex, Kobe, Japan). ALP, gamma-GT, AST, ALT, total bilirubin, total protein, albumin, BUN, creatinine, glucose, creatine kinase, and LDH levels were measured with an ADVIA^®^ 2400 chemistry system (Siemens, Bayern, Germany).

### Evaluation of diet and physical activity

Three-day food records and physical activity records were collected at each visit to evaluate participants’ usual diet and physical activity patterns. A single dietitian analyzed the dietary intake data using CAN-pro 4.0 software (The Korean Nutrition Society, Seoul, Korea), and physical activity was evaluated with the Global Physical Activity Questionnaire (GPAQ).

### Statistical analysis

Statistical analyses were performed with SAS software, version 9.4 (SAS Institute Inc., Charlotte, NC, USA). Data are presented as the mean ± standard deviation (SD). The study was designed to detect a 1.4-kg (SD = 2.0 kg) difference in body fat mass between the groups with 80% power and a two-tailed α-value of 0.05 (Grube et al., 2013). The appropriate sample size for each group in the study was determined to be 32 participants, allowing for a 20% dropout rate. The intention-to-treat (ITT) analysis included all randomized participants who received at least one dose of the SGE or placebo supplement.

The efficacy and safety parameters of the ITT group were analyzed. A Chi square test was performed to determine differences in the frequencies of categorical variables between the groups. A linear mixed-effects model was applied to repeated-measures data for each continuous outcome. The fixed effects were treatment group, treatment visit, and the interaction between treatment group and visit. A *p* value < 0.05 was considered statistically significant.

## Results and discussion

### Participants

In total, 80 female participants were enrolled in this study and were equally divided into the SGE and placebo groups. Four participants from the SGE group and six from the placebo group withdrew from the study for personal reasons. Ultimately, 70 participants (36 SGE and 34 placebo) completed the study. The demographic characteristics of the participants are shown in Table 2. There were no significant differences between the SGE and placebo groups in baseline characteristics such as age, anthropometric parameters, and vital signs.

**Table 2:** Demographic characteristics of the study subjects

	Placebo group (n = 40)	SGE group (n = 40)	*p* value^a^
Age (years)	33.58 ± 9.80	32.38 ± 9.44	0.579
Height (cm)	161.38 ± 4.40	161.95 ± 6.23	0.635
Weight (kg)	69.54 ± 3.86	71.50 ± 7.35	0.142
BMI (kg/m^2^)	26.71 ± 1.51	27.20 ± 1.64	0.168
SBP (mmHg)	117.93 ± 10.28	116.60 ± 13.36	0.621
DBP (mmHg)	75.63 ± 9.06	74.43 ± 10.50	0.586
Pulse (BPM)	78.23 ± 10.56	76.23 ± 8.16	0.346

Values are presented as mean ± SD

*BMI* body mass index, *SBP* systolic blood pressure, *DBP* diastolic blood pressure

^a^Analyzed by independent *t* test

### Safety evaluation

Three participants experienced adverse events during the study period: one in the SGE group and two in the placebo group. The adverse events reported in the SGE group were common cold and dyspepsia. The difference between the two groups in terms of adverse events was not significant. Moreover, none of the adverse events were deemed by the study investigators to be SGE-related. The evaluation was also expanded to include laboratory tests, electrocardiography, and vital signs measurements. All clinical test results were in the normal range, and no participant withdrew because of adverse events.

### Diet and physical activity

No significant differences in dietary intake (calories, carbohydrates, protein, fat, and fiber) or physical activity (metabolic equivalents value) were observed between the groups during the intervention period (Table 3).

**Table 3:** Major nutrient intakes and metabolic equivalent values of the SGE and placebo groups measured at 0 and 12 weeks

	Placebo group (n = 40)			SGE group (n = 40)			*p* value^b^
	0 weeks	12 weeks	*p* value^a^	0 weeks	12 weeks	*p* value^a^	
Major nutrient intakes							
Calories (kcal)	1549.11 ± 501.82	1442.89 ± 347.85	0.207	1593.68 ± 445.97	1505.31 ± 439.22	0.084	0.964
Carbohydrates (g)	217.83 ± 79.58	212.54 ± 63.56	0.684	223.84 ± 64.02	210.62 ± 69.67	0.252	0.596
Lipids (g)	47.18 ± 16.30	41.10 ± 15.01	0.085	47.50 ± 20.07	46.21 ± 19.57	0.604	0.383
Protein (g)	58.21 ± 18.29	52.28 ± 14.21	0.111	60.33 ± 20.12	57.51 ± 19.53	0.377	0.618
Fiber (g)	15.40 ± 5.68	15.06 ± 5.23	0.765	16.09 ± 5.80	15.28 ± 5.50	0.299	0.542
Metabolic equivalent values							
MET value (min/week)	2644.50 ± 5070.78	1608.89 ± 2523.55	0.179	2655.50 ± 5348.13	1768.33 ± 2855.48	0.173	0.908

Values are presented as mean ± SD

*MET* metabolic equivalent

^a^Analyzed by a linear mixed model (differences within a group)

^b^Analyzed by a linear mixed model (differences between groups)

### Changes in anthropometric parameters

After the 12-week intervention, mean body weight and BMI decreased in the SGE group, while these parameters increased in the placebo group, with significant differences between the two groups (*p *= 0.047 and *p *= 0.035, respectively). The differences between the SGE and placebo groups were ~ 1.5% for body weight and ~ 0.5% for the BMI. However, there was no significant change in waist or hip circumference in either group (Table 4).

**Table 4:** Anthropometric parameters of the SGE and placebo groups measured at 0 and 12 weeks

	Placebo group (n = 40)			SGE group (n = 40)			*p* value^b^
	0 weeks	12 weeks	*p* value^a^	0 weeks	12 weeks	*p* value^a^	
Weight (kg)	69.54 ± 3.86	70.09 ± 4.56	0.282	71.50 ± 7.35	70.51 ± 6.89	0.098	0.047
BMI (kg/m^2^)	26.71 ± 1.51	26.95 ± 1.96	0.210	27.20 ± 1.64	26.83 ± 1.84	0.096	0.035
WC (cm)	90.66 ± 4.56	90.54 ± 4.84	0.403	91.79 ± 5.73	90.99 ± 5.33	0.086	0.571
HC (cm)	100.86 ± 3.51	100.84 ± 3.40	0.505	101.71 ± 5.23	101.25 ± 4.92	0.159	0.567

Values are presented as mean ± SD

*BMI* body mass index, *WC* waist circumference, *HC* hip circumference

^a^Analyzed by a linear mixed model (differences within a group)

^b^Analyzed by a linear mixed model (differences between groups)

### Changes in body composition

After the 12-week intervention, fat mass in the arms decreased in the SGE group but increased in the placebo group, with significant difference between the two groups [left (*p *= 0.018), right (*p *= 0.030), and total (*p *= 0.012)]. Percent body fat in the arms tended to decrease more in the SGE group than in the placebo group, though the differences were not significant [left (*p *= 0.051) and right (*p *= 0.057)]. The sum of the percent body fat in both arms decreased in the SGE group but increased in the placebo group, and there was a statistically significant difference between the two groups (*p *= 0.035). Body fat mass and percent body fat in both legs did not differ between the two groups. Trunk body fat mass and percent body fat decreased significantly in both groups, with no statistically significant difference between the groups; unexpectedly, the decrease was greater in the placebo group. In the SGE group, the total body fat mass and percent body fat decreased by about 3.38% (*p *= 0.026) and 2.51% (*p *= 0.020), respectively, but there were no statistically significant differences compared to the placebo group (Table 5).

**Table 5:** Body composition of the SGE and placebo groups measured at 0 and 12 weeks

	Placebo group (n = 40)			SGE group (n = 40)			*p* value^b^
	0 weeks	12 weeks	*p* value^a^	0 weeks	12 weeks	*p* value^a^	
*Left arm*							
BFM (g)	1917.32 ± 412.52	2078.81 ± 464.39	0.061	2097.74 ± 620.98	1956.16 ± 513.48	0.128	0.018
%BF (%)	48.89 ± 5.13	50.03 ± 5.10	0.152	48.75 ± 5.44	47.48 ± 5.22	0.175	0.051
*Right arm*							
BFM (g)	1924.77 ± 436.18	2123.56 ± 434.24	0.037	2232.97 ± 650.45	2141.61 ± 462.15	0.370	0.030
%BF (%)	47.74 ± 5.55	49.69 ± 4.97	0.028	48.47 ± 6.25	47.78 ± 5.19	0.761	0.057
*Arms*							
BFM (g)	3842.10 ± 821.24	4202.36 ± 815.95	0.032	4330.70 ± 1227.26	4097.78 ± 912.90	0.152	0.012
%BF (%)	48.31 ± 5.19	49.91 ± 4.74	0.038	48.62 ± 5.68	47.68 ± 4.99	0.369	0.035
*Left leg*							
BFM (g)	4263.87 ± 605.54	4332.63 ± 704.42	0.387	4236.16 ± 972.15	4168.19 ± 861.00	0.803	0.482
%BF (%)	39.11 ± 4.28	39.49 ± 4.35	0.637	37.94 ± 4.63	37.04 ± 4.50	0.208	0.196
*Right leg*							
BFM (g)	4228.89 ± 598.57	4282.86 ± 649.93	0.673	4195.00 ± 828.49	4119.52 ± 858.06	0.612	0.508
%BF (%)	38.93 ± 4.24	39.22 ± 4.52	0.892	37.61 ± 4.55	36.86 ± 4.58	0.350	0.416
*Legs*							
BFM (g)	8492.76 ± 1177.18	8615.49 ± 1323.37	0.473	8431.15 ± 1784.98	8287.72 ± 1693.84	0.697	0.465
%BF (%)	39.02 ± 4.21	39.36 ± 4.34	0.769	37.77 ± 4.54	37.76 ± 3.34	0.266	0.285
Trunk							
BFM (g)	13,001.13 ± 2347.47	12,361.88 ± 2053.55	0.001	12,636.05 ± 2503.77	11,865.17 ± 2689.20	0.023	0.905
%BF (%)	38.44 ± 5.43	36.54 ± 4.58	0.005	37.03 ± 4.70	35.19 ± 5.56	0.013	0.598
*Total*							
BFM (g)	26,181.26 ± 3419.68	25,980.46 ± 3119.89	0.200	26,307.39 ± 4661.40	25,127.44 ± 4473.13	0.026	0.286
%BF (%)	38.93 ± 4.02	38.43 ± 3.54	0.067	38.00 ± 3.86	36.69 ± 4.12	0.020	0.629

Values are presented as mean ± SD

*BFM* body fat mass, *%BF* percent body fat

^a^Analyzed by a linear mixed model (differences within a group)

^b^Analyzed by a linear mixed model (differences between groups)

### Changes in blood markers

After the 12-week intervention, adiponectin level in the SGE group was reduced (*p *= 0.034) but did not differ significantly from that of the placebo group. There were no significant changes in other blood markers, including leptin (Table 6).

**Table 6:** Blood markers of the SGE and placebo groups measured at 0 and 12 weeks

	Placebo group (n = 40)			SGE group (n = 40)			*p* value^b^
	0 weeks	12 weeks	*p* value^a^	0 weeks	12 weeks	*p* value^a^	
TC (mg/dL)	190.78 ± 31.45	196.00 ± 34.51	0.304	184.40 ± 25.14	180.75 ± 27.35	0.584	0.254
TG (mg/dL)	103.05 ± 45.10	113.19 ± 72.30	0.418	105.43 ± 59.55	110.78 ± 69.82	0.570	0.871
LDL-C (mg/dL)	111.93 ± 27.54	115.67 ± 28.71	0.351	106.15 ± 23.56	103.50 ± 22.75	0.648	0.281
HDL-C (mg/dL)	58.20 ± 12.23	58.28 ± 13.75	0.978	57.10 ± 11.91	55.14 ± 9.51	0.371	0.504
Adiponectin (μg/mL)	8.49 ± 2.65	8.18 ± 2.87	0.458	8.41 ± 3.12	7.46 ± 2.69	0.034	0.173
Leptin (ng/mL)	30.23 ± 10.77	36.81 ± 12.89	0.002	31.08 ± 14.33	30.52 ± 15.14	0.939	0.053

Values are presented as mean ± SD

*TC* total cholesterol, *TG* triglycerides, *LDL*-*C* low-density lipoprotein cholesterol, *HDL*-*C* high-density lipoprotein cholesterol

^a^Analyzed by a linear mixed model (differences within a group)

^b^Analyzed by a linear mixed model (differences between groups)

### Discussion

Ginger is a perennial plant that has been used for centuries as a spice and in folk medicine. The pharmacological properties of ginger are mainly attributed to 6-gingerol and 6-shogaol (Semwal et al., 2015). Of the two, 6-gingerol is mainly present in fresh ginger and has a β-hydroxy-ketone skeleton. Since its structure is thermally unstable, 6-gingerol is converted to 6-shogaol, which has an α,β-unsaturated carbonyl group, as a result of processing techniques such as heating, drying, and steaming (Bhattarai et al., 2001; 2007; Dugasani et al., 2010; Kou et al., 2018). Both substances are beneficial to health, but several recent reports have indicated that 6-shogaol is superior to 6-gingerol (Dugasani et al., 2010; Ho and Chang, 2018; Jin et al., 2014; Wang et al., 2017), presumably due to its α,β-unsaturated carbonyl group (Kou et al., 2018). An α,β-unsaturated carbonyl group is present not only in 6-shogaol, but also in curcumin and chalcone (Rodrigues et al., 2016), which are known to have excellent pharmacological activities. In the chalcone family, licochalcone A is present in licorice and is known to have antioxidant (Lv et al., 2015), anti-inflammatory (Jia et al., 2017; Kim et al., 2010), anti-allergic (Majima et al., 2004), and neuroprotective (Huang et al., 2017; Liu et al., 2018) effects. According to Megumi et al. (2010), licochalcone A has anti-inflammatory effects, while reduced licochalcone A, which lacks a double bond, fails to inhibit inflammatory reactions. This difference is probably due to the fixed planar conformation resulting from the double bonds. In addition, heat-treated licorice extract was reported to have greater beneficial effects than licorice extract (Kim et al., 2010). Although further studies are needed to determine the exact mechanism, 6-shogaol might be more potent than other substances in ginger due to the double bond in its α,β-unsaturated carbonyl group.

SGE is an ethanol extract of steamed ginger and has a greater 6-shogaol content than other ginger products. According to Jung et al. (Jung et al., 2018), moist heat treatment yields more 6-shogaol than dry heat. It is known that 6-shogaol has health benefits, and quality control measures can be performed more reliably with this compound than with 6-gingerol. So far, various beneficial effects of ginger have been studied, but research on SGE has been limited to preclinical studies. This study was a 12-week, randomized, double-blind, placebo-controlled clinical trial to evaluate the efficacy and safety of 6-shogaol-enriched SGE for weight and body fat loss in obese Korean participants. Thus, this study was the first to evaluate the efficacy of SGE in humans. In this study, body weight, BMI, and body fat in the arms were significantly reduced after 12 weeks of SGE supplementation.

Although we did not provide strong evidence of the anti-obesity effects of SGE, our findings are partly supported by the existing in vitro and in vivo studies. In 3T3-L1 adipocytes, SGE significantly reduced the expression of master regulators of adipogenesis such as *Pparg* and *Cebpa* and thus suppressed the expression of lipogenesis- and insulin-sensitivity-related genes such as *Fabp4*, *Slc2a4*, *Fasn*, *Acaca*, and *Adipoq* (unpublished data). These results indicate that SGE effectively inhibits adipocyte differentiation and lipid accumulation. Additionally, in mice fed a high-fat diet, SGE supplementation attenuated increases in body weight, body fat, blood lipid levels, and hepatic TG accumulation. These effects were thought to be related to a decrease in SREBP1c (*Srebf1*) expression. Moreover, SGE supplementation was reported to increase the adiponectin concentration, known as obesity-related adipokine, in mice (Kim et al., 2018). Thus, SGE supplementation seems to have anti-obesity effects in human subjects; however, larger-scale studies are needed to verify this conclusion.

There were two reported adverse events for one participant in the SGE group in this clinical trial. However, the incidence of adverse events did not differ significantly between the two groups, and the adverse events were not related to SGE intake. Also, there were no clinically significant changes in laboratory tests, electrocardiogram findings, or vital signs. Ginger is a food and medicinal ingredient that has a long history and is generally considered safe. It is also listed on the Generally Recognized as Safe (GRAS) list by the United States Food and Drug Administration (US FDA), including during lactation (O’Hara et al., 1998), suggesting that SGE is a safe supplement.

A potential limitation of this study is that all the participants were women. In general, women have a higher percentage of body fat than men. The prevalence of obesity is also known to be higher in women than in men (Kanter and Caballero, 2012; Park et al., 2003). Female participants were mainly selected for these reasons; however, to generalize the effects of SGE, further studies with male participants are needed.

In conclusion, our results suggest that SGE supplementation has beneficial effects on certain markers of body weight and body fat. However, this study was conducted to evaluate the effects of supplementation without dietary control or increased physical activity, and the anti-obesity effects of SGE alone may be insufficient. Previous studies have demonstrated that ginger supplementation is more effective when combined with physical activity (Ayaz and Roshan, 2012; Khosravani et al., 2016). Therefore, 6-shogaol-enriched SGE supplementation combined with lifestyle modifications such as diet control and/or physical activity might have greater potential for weight and body fat reduction. Also, since the analysis of SGE is still in its early stages, additional well-designed studies are needed.

## Electronic supplementary material

Below is the link to the electronic supplementary material.

Supplementary material 1 (DOCX 60 kb)
