# Supplementary material for: The effects of steamed ginger ethanolic extract on weight and body fat loss: a randomized, double-blind, placebo-controlled clinical trial
Source: Food Sci Biotechnol. 2019 Oct 11;29(2):265–73. doi: 10.1007/s10068-019-00649-x (PMC6992804; doi:10.1007/s10068-019-00649-x)
Supplement: Supplementary file 1 — Supplementary material 1 (DOCX 60 kb) [file 10068_2019_649_MOESM1_ESM.docx]

**Figure caption for Supplemental Figure**

**Figure S1**. **Representative HPLC chromatogram of SGE.** 6-shogaol (retention time: 24.2 min) was analyzed with a Waters Symmetry C18 5 μm column (2.1 × 150 mm) at 230 nm under a gradient condition of A solution (0.005% formic acid in water) and B solution (0.005% formic acid in acetonitrile). The elution gradient was as follows: 0-2 min, 20-20% (B%); 2-25 min, 20-60% (B%); 25-40 min, 60-100% (B%); 40-45 min, 100-100% (B%).


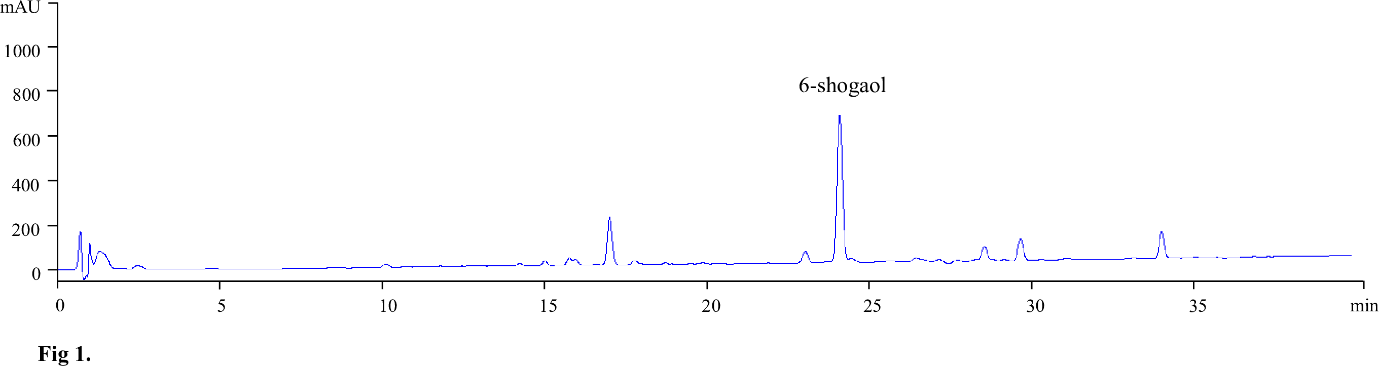


**Fig S1**.
